# Effectiveness of a hybrid emergency room system in the management of acute ischemic stroke: a single-center experience

**DOI:** 10.3389/fmed.2024.1420951

**Published:** 2024-07-04

**Authors:** Masahiro Kashiura, Chisato Nakajima, Yuki Kishihara, Keiichiro Tominaga, Hiroyuki Tamura, Hideto Yasuda, Masashi Ikota, Kenji Yamada, Yoshikazu Yoshino, Takashi Moriya

**Affiliations:** ^1^Department of Emergency and Critical Care Medicine, Saitama Medical Center, Jichi Medical University, Saitama, Japan; ^2^Department of Endovascular Surgery, Saitama Medical Center, Jichi Medical University, Saitama, Japan; ^3^Department of Neurosurgery, Saitama Medical Center, Jichi Medical University, Saitama, Japan

**Keywords:** cerebral infarction, endovascular procedures, intracranial embolism and thrombosis, radiographic image enhancement, thrombectomy

## Abstract

**Introduction:**

Hybrid emergency room systems (HERSs) have shown promise for the management of severe trauma by reducing mortality. However, the effectiveness of HERSs in the treatment of acute ischemic stroke (AIS) remains unclear. This study aimed to evaluate the impact of HERSs on treatment duration and neurological outcomes in patients with AIS undergoing endovascular therapy.

**Materials and methods:**

This single-center retrospective study included 83 patients with AIS who were directly transported to our emergency department and underwent endovascular treatment between June 2017 and December 2023. Patients were divided into the HERS and conventional groups based on the utilization of HERSs. The primary outcome was the proportion of patients achieving a favorable neurological outcome (modified Rankin Scale score 0–2) at 30 days. The secondary outcomes included door-to-puncture and door-to-recanalization times. Univariate analysis was performed using the Mann–Whitney U test for continuous variables and the chi-squared test or Fisher’s exact test for categorical variables, as appropriate.

**Results:**

Of the 83 eligible patients, 50 (60.2%) were assigned to the HERS group and 33 (39.8%) to the conventional group. The median door-to-puncture time was significantly shorter in the HERS group than in the conventional group (99.5 vs. 131 min; *p* = 0.001). Similarly, the median door-to-recanalization time was significantly shorter in the HERS group (162.5 vs. 201.5 min, *p* = 0.018). Favorable neurological outcomes were achieved in 16/50 (32.0%) patients in the HERS group and 6/33 (18.2%) in the conventional group. The HERS and conventional groups showed no significant difference in the proportion of patients achieving favorable neurological outcomes (*p* = 0.21).

**Conclusion:**

Implementation of the HERS significantly reduced the door-to-puncture and door-to-recanalization times in patients with AIS undergoing endovascular therapy. Despite these reductions in treatment duration, no significant improvement in neurological outcomes was observed. Further research is required to optimize patient selection and treatment strategies to maximize the benefits of the HERS in AIS management.

## 1 Introduction

Acute ischemic stroke (AIS) caused by large-vessel occlusion is associated with poor outcomes. In cases of ischemic stroke, mortality within 3–6 months was higher after large-vessel occlusion than after non-large-vessel occlusion (26.2% vs. 1.3%, odds ratio: 4.1, 95% confidence interval: 2.5–6.7) (1). The penumbra, the tissue surrounding the infarction area that is at risk of cell death, can be salvaged by prompt recanalization (2). Numerous randomized trials have demonstrated the advantages of endovascular treatment, including thrombectomy following intravenous thrombolysis with a tissue plasminogen activator (tPA), in achieving recanalization and favorable neurological outcomes (3). Early successful recanalization after symptom onset remains a critical factor for optimal outcomes (4).

The implementation of hybrid emergency room (ER) systems (HERSs) in Japan has shown promising results in the management of cases involving severe trauma. An HERS is equipped with advanced diagnostic modalities, such as X-ray fluoroscopy and computed tomography (CT), enabling complete diagnosis and immediate treatment without the need for patient transfer (5, 6). The consequent elimination of transfer time has led to a decrease in mortality rates in patients with trauma (7). Timely reperfusion is crucial in AIS caused by large-vessel occlusion (8). Given the critical importance of time in both severe trauma and AIS management, the HERS could plausibly provide similar benefits for stroke care (9). However, the effectiveness of the HERS in the management of AIS remains unclear.

Therefore, this study aimed to retrospectively analyze patients who underwent endovascular treatment for AIS at our institution and evaluate the effectiveness of the HERS in this context. We hypothesized that the use of the HERS, which enables immediate CT diagnosis and rapid endovascular intervention without patient transfer, would result in shorter time intervals from hospital arrival to recanalization and, consequently, yield improved neurological outcomes in comparison with conventional stroke management. By addressing this gap in the current understanding of HERS applications, we aimed to contribute to the advancement of stroke care and potentially offer insights into the optimization of ER systems for the management of AIS.

## 2 Materials and methods

### 2.1 Study design and setting

**Figure 1:**
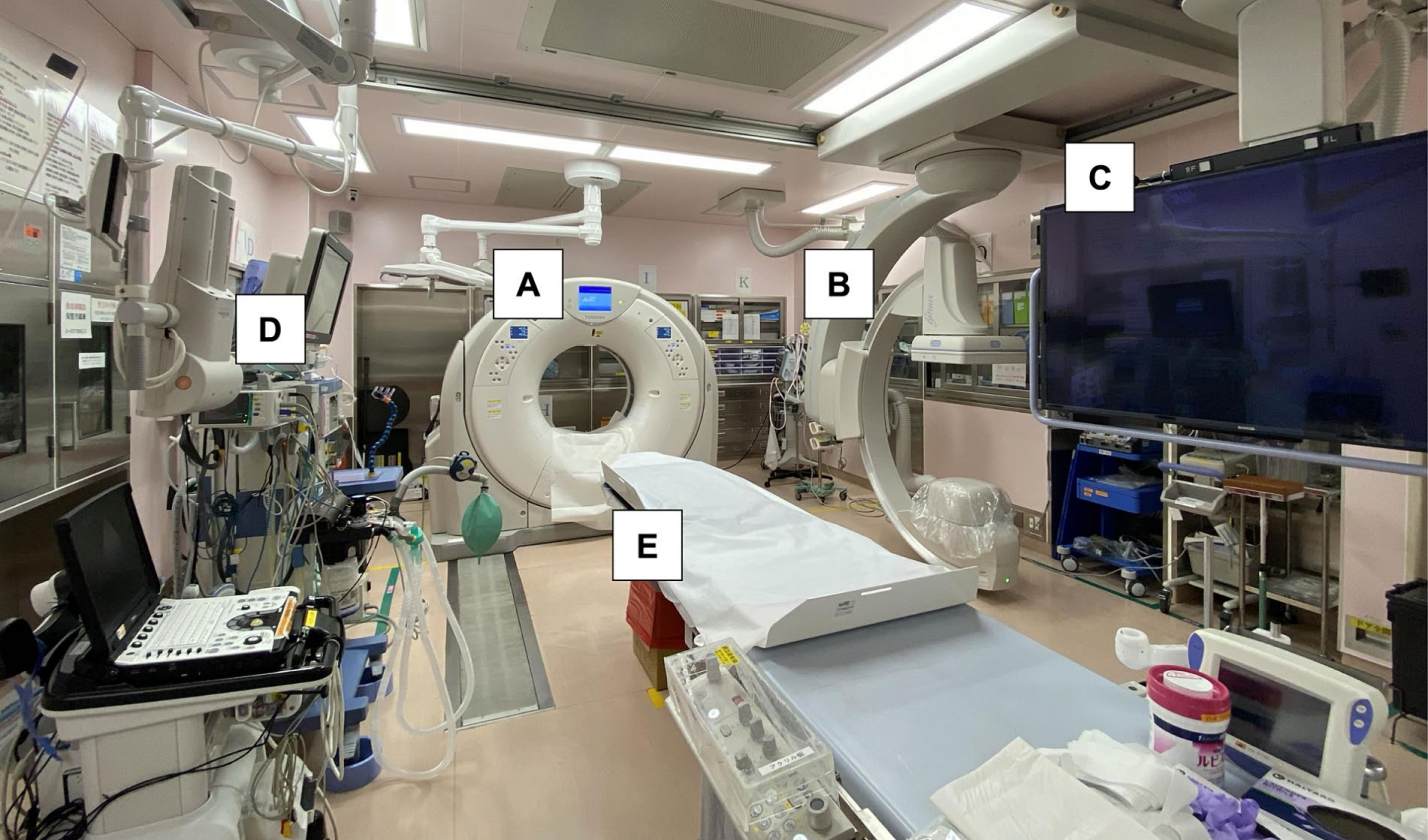
Photograph showing a sliding computed tomography scanner system with interventional radiology features in the emergency room. Acute procedures for ischemic stroke, including airway management, and angiography can be performed on the table without transferring the patient. **(A)** Sliding computed tomography (CT) scan device, **(B)** movable C-arm, **(C)** monitoring screen, **(D)** mechanical ventilator with anesthesia apparatus, and **(E)** CT examination and intervention table.

This retrospective study was conducted at a single academic medical center located in an urban area of Kanto, Japan. The HERS, which was equipped with a sliding CT scanner system featuring interventional radiology capabilities, was established adjacent to the conventional ER in 2016 (Figure 1). The distance from the ambulance parking lot to the conventional ER and the hybrid ER was approximately 50 m. Acute AIS management using the HERS commenced in 2017, following the assignment of neuroendovascular specialists.

The HERS was preferentially employed when a patient with suspected stroke was transported within 6 h of symptom onset. All patients who were transported to our emergency department with suspected acute stroke underwent an initial evaluation by an emergency physician; if the hybrid ER was available, the patients were treated using the hybrid ER immediately after transport, and if the hybrid ER was being used to treat other patients, the patients were treated in the conventional ER. AIS was diagnosed using head CT, including perfusion imaging, or head magnetic resonance imaging in patients with renal dysfunction. Thrombolytic therapy was administered by the emergency physicians in consultation with a stroke specialist. Endovascular treatment was administered to patients with onset within 6 h or an unknown onset time showing occlusion of the internal carotid artery, M1 or M2 portion of the middle cerebral artery, or basilar artery along with a relatively large penumbra compared to the ischemic core, as judged by the emergency physician. An endovascular physician performed all endovascular treatments.

This study was conducted in accordance with the Strengthening the Reporting of Observational Studies in Epidemiology (STROBE) statement (Supplementary material File 1) (10). This study was approved by the Institutional Review Board of Jichi Medical University Saitama Medical Center. Participants were provided with the opportunity to opt out of the study at any time by withdrawing permission to use their data.

### 2.2 Patient selection and grouping

This investigation included a cohort of patients with AIS who were directly transported from prehospital locations to our emergency department by emergency medical service personnel and received endovascular treatment between June 2017 and December 2023. Patients who developed AIS at our hospital or who were transferred from other hospitals or clinics were excluded.

The study population was divided into two groups: the HERS group, in which patients received continuous care from initial evaluation to endovascular treatment using the HERS, and the conventional group, in which the HERS was not used.

### 2.3 Data collection

The following data were collected from electronic medical records: age, sex, modified Rankin Scale (mRS) score before admission (11), onset-to-door time (interval from symptom onset to hospital arrival), National Institutes of Health Stroke Scale (NIHSS) score on admission (12), door-to-picture time (interval from hospital arrival to CT imaging), Alberta Stroke Program Early Computed Tomography Score (ASPECTS) on admission (13), etiology of AIS (atrial fibrillation, atherothrombotic, left-to-right shunt, or cryptogenic), culprit lesion (M1 or M2 segment of middle cerebral artery, internal carotid artery, basilar artery), presence of tandem lesions, tPA administration, door-to-needle time (interval from hospital arrival to tPA administration), endovascular treatment procedure (fragmentation, mechanical thrombectomy, or carotid artery stenting and mechanical thrombectomy), door-to-puncture time (interval from hospital arrival to arterial puncture), door-to-recanalization time (interval from hospital arrival to recanalization), onset-to-recanalization time (interval from symptom onset to recanalization), Thrombolysis in Cerebral Infarction (TICI) grade, intracranial hemorrhage after procedure (symptomatic or asymptomatic), ASPECTS 30 days after admission, length of hospital stay, NIHSS score 30 days after admission, and mRS score 30 days after admission.

### 2.4 Outcome measures

The primary outcome was a favorable neurological outcome at 30 days, defined as an mRS score of 0–2. The secondary outcomes were door-to-puncture and door-to-recanalization times as direct measures of time reduction using the HERS, in addition to the distribution of the mRS score at 30 days.

### 2.5 Statistical analyses

Descriptive statistics were calculated for all the variables of interest. Continuous variables were presented as median and interquartile range (IQR), whereas categorical variables were presented as count and percentage. Univariate analysis was performed using the Mann–Whitney U test for continuous variables and the chi-squared test or Fisher’s exact test for categorical variables, as appropriate.

All statistical tests were two-sided, and *p*-values <0.05 were considered statistically significant. Statistical analyses were performed using R Statistical Software.

## 3 Results

### 3.1 Patient enrollment and grouping of eligible patients

**Figure 2:**
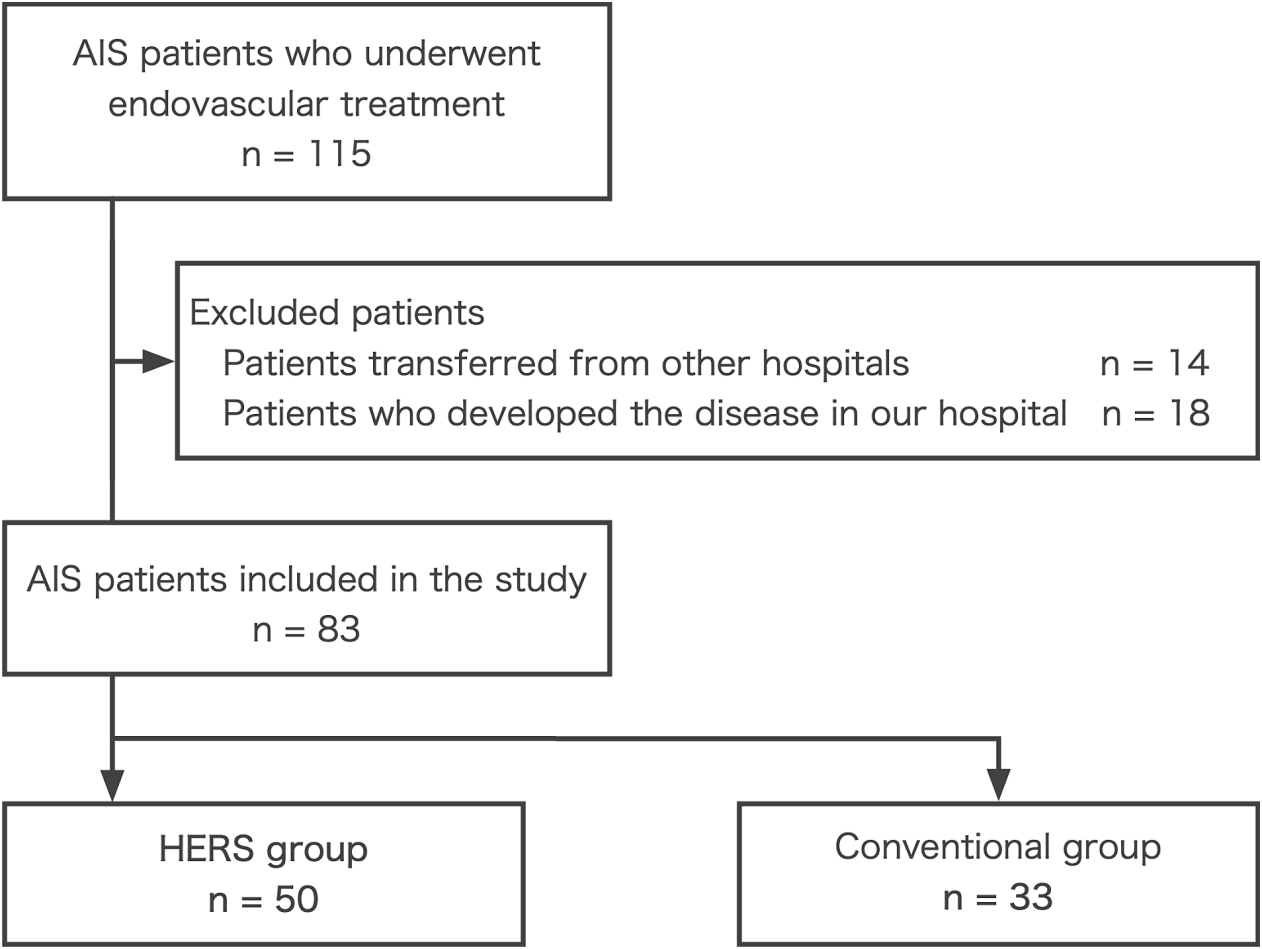
Patient selection flowchart. AIS, acute ischemic stroke; HERS, hybrid emergency room system.

During the study period, 115 patients with AIS underwent endovascular treatment. Of these, 14 patients (12.2%) were transferred from other hospitals, and 18 (15.7%) developed AIS at our hospital (Figure 2). After excluding these patients, 83 patients with AIS who were directly transported from prehospital locations to the emergency department and underwent endovascular treatment were included in the study. Of these, 50 patients (60.2%) were treated with the HERS (HERS group), whereas 33 (39.8%) were treated in the conventional ER (conventional group).

### 3.2 Patient characteristics

There were no missing data. The patient demographics, clinical characteristics, and outcomes are summarized in Table 1. The median age of the study population was 79 years (IQR: 73–85 years), and 62.7% of the patients were male. The median onset-to-door time was 55 min (IQR, 41–111 min), and the median NIHSS score at admission was 20 (IQR: 13.5–28). The most common etiology of AIS was atrial fibrillation (67.5%), and the culprit lesions were most frequently present in the M1 segment of the middle cerebral artery (53.0%). Tandem lesions were present in 4.8% of the patients. tPA was administered in 67.5% of the cases.

**Table 1:** Patient demographics, characteristics, and outcomes.

Factor	Overall (*n* = 83)	HERS group (*n* = 50)	Conventional group (*n* = 33)	*p* value
Age, years	79 [73–85]	78.5 [72.5–84]	79 [73–86]	0.62
Male (%)	52 (62.7)	31 (62.0)	21 (63.6)	1
mRS score before admission	0 [0–1]	0 [0–1]	0 [0–1]	0.74
Onset-to-door time, min	55 [41–111]	55 [41.5–80]	56 [41–157]	0.53
NIHSS score at admission	20 [13.5–28]	20 [11.5–29]	21 [14–26]	0.87
Door-to-picture time, min	20 [15.5–28]	19 [13–23]	28 [17–40]	<0.001
ASPECTS at admission	9 [8–10]	9 [8–10]	9 [8–10]	0.64
Etiology (%)				
Cryptogenic	20 (24.1)	8 (16.0)	12 (36.4)	0.13
Atrial fibrillation	56 (67.5)	38 (76.0)	18 (54.5)	
Arteriosclerotic disease	5 (6.0)	3 (6.0)	2 (6.1)	
Right-to-left shunt	2 (2.4)	1 (2.0)	1 (3.0)	
Culprit lesion (%)				
M1 segment of middle cerebral artery	44 (53.0)	29 (58.0)	15 (45.5)	0.51
M2 segment of middle cerebral artery	12 (14.5)	6 (12.0)	6 (18.2)	
Internal carotid artery	20 (24.1)	10 (20.0)	10 (30.3)	
Basilar artery	7 (8.4)	5 (10.0)	2 (6.1)	
Tandem lesions (%)	4 (4.8)	2 (4.0)	2 (6.1)	1
Tissue plasminogen activator administration (%)	56 (67.5)	37 (74.0)	19 (57.6)	0.15
Door-to-needle time, min	77 [66.5–100.5]	74 [64–84]	100 [78–107]	0.007
Procedures (%)				
Fragmentation	2 (2.4)	0 (0.0)	2 (6.1)	0.16
Mechanical thrombectomy	80 (96.4)	49 (98.0)	31 (93.9)	
Carotid artery stenting and mechanical thrombectomy	1 (1.2)	1 (2.0)	0 (0.0)	
Door-to-puncture time, min	107 [83–132]	99.5 [75.75–117.5]	131 [98–166]	0.001
Door-to-recanalization time, min	170 [142–216.5]	162.5 [141.75–184.5]	201.5 [154–251]	0.018
Onset-to-recanalization time, min	239 [190.5–318]	225 [188.25–269.75]	274 [201–369]	0.10
Thrombolysis in cerebral infarction grade (%)				
0	2 (2.4)	2 (4.0)	0 (0.0)	0.34
1	2 (2.4)	0 (0.0)	2 (6.1)	
2a	10 (12.0)	6 (12.0)	4 (12.1)	
2b	24 (28.9)	13 (26.0)	11 (33.3)	
3	45 (54.2)	29 (58.0)	16 (48.5)	
Intracranial cerebral hemorrhage (%)	16 (19.3)	9 (18.0)	7 (21.2)	0.78
Symptomatic intracranial cerebral hemorrhage (%)	11 (13.3)	6 (12.0)	5 (15.2)	0.75
ASPECTS 30 days after admission	7 [5–8.5]	6 [5–8]	7 [4–9]	0.77
Length of hospital stay, days	23 [14.5–31]	22.5 [14.5–30.5]	23 [15–31]	0.71
NIHSS score 30 days after admission	16 [8.5–27]	16 [9–27]	18 [7–26]	0.94
mRS score 30 days after admission	4 [2–5]	4 [2–5]	4 [3–5]	0.22
Favorable neurological outcome 30 days after admission (%)	22 (26.5)	16 (32.0)	6 (18.2)	0.21

Continuous variables are presented as median [interquartile range]. Categorical variables were presented as count (percentage). ASPECTS, Alberta Stroke Program Early Computed Tomography Score; HERS, hybrid emergency room system; NIHSS, National Institutes of Health Stroke Scale; mRS, modified Rankin Scale.

### 3.3 Patient outcomes

The median door-to-puncture time was significantly shorter in the HERS group than in the conventional group (99.5 [IQR: 75.75–117.5] min vs. 131 [IQR: 98–166] min, *p* = 0.001). Similarly, the median door-to-recanalization time was significantly shorter in the HERS group (162.5 [IQR: 141.75–184.5] min vs. 201.5 [IQR: 154–251] min, *p* = 0.018). However, the onset-to-recanalization time was not significantly different between the two groups (225 [IQR: 188.25–269.75] min vs. 274 [201–369] min, *p* = 0.10).

**Figure 3:**
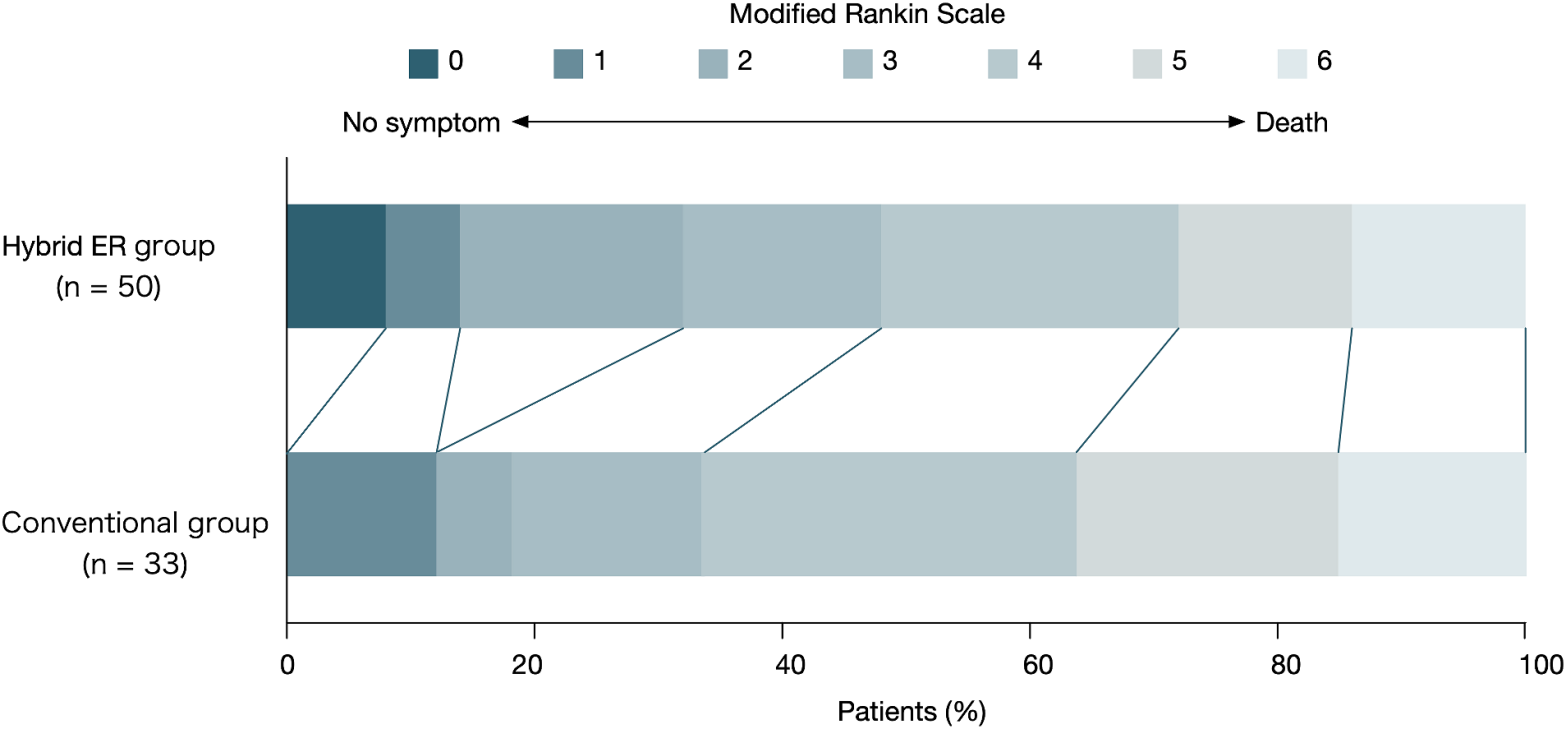
Distribution of modified Rankin Scale (mRS) scores at 30 days.

The primary outcome, i.e., an mRS score of 0–2 at 30 days after admission, was achieved in 32.0% of the patients in the HERS group and 18.2% of those in the conventional group; however, this difference was not statistically significant (*p* = 0.21). The distribution of the mRS scores 30 days after admission is shown in Figure 3.

## 4 Discussion

This retrospective study aimed to evaluate the efficacy of the HERS in managing patients with AIS undergoing endovascular treatment. Our findings demonstrate that the implementation of the HERS significantly reduced the median door-to-puncture and door-to-recanalization times in comparison with the conventional approach. However, despite the observed reduction in treatment times, the HERS and conventional groups showed no significant difference in the proportion of patients achieving favorable neurological outcomes. The significant reduction in door-to-puncture and door-to-recanalization times associated with the HERS highlights the potential of this novel system for streamlining the care pathway for patients with AIS.

By enabling rapid diagnosis and treatment initiation without the need for patient transfer, the HERS can minimize the delays that often compromise the effectiveness of endovascular therapy (9). The significant reduction in the treatment time observed in the HERS group in this study was consistent with the findings of previous studies demonstrating the benefits of the HERS in the management of acute conditions (14, 15). Kinoshita et al. found that the use of a hybrid ER significantly reduced the time required for CT examinations and emergency surgery in patients with severe traumatic brain injury (16). These findings support the notion that the integrated imaging and intervention capabilities of the HERS can streamline the care pathway for acute conditions and eliminate delays associated with patient transfer (6). Because early successful recanalization is a critical factor for favorable outcomes in AIS (2), the time savings afforded by the HERS may have important implications for patient prognosis.

However, the lack of a significant difference in favorable neurological outcomes between the HERS and conventional groups warrants further discussion. Although the use of the HERS significantly reduced treatment time, it did not translate into improved neurological outcomes in our study population. Expanding the application of the HERS to a broader range of patients with AIS may lead to improved outcomes; however, this requires careful consideration and further research to identify optimal patient selection criteria. Furthermore, the median difference in the door-to-recanalization time to reperfusion was <40 min, and the onset-to-recanalization time was not significantly different, suggesting that the prognostic impact may be limited.

Current trends and future directions in AIS treatment emphasize the importance of reducing door-to-groin time and improving outcomes by employing a direct-to-angiography suite approach in the early time window for selected patients with large-vessel occlusions (17). Although our study demonstrated the effectiveness of the HERS in reducing treatment time, the lack of improvement in neurological outcomes highlights the need for further optimization of HERS-based treatment strategies. This may involve refining patient selection criteria, improving device selection and technical aspects of the procedure, and ensuring seamless coordination among multidisciplinary stroke teams.

This study had several limitations. First, the door-to-puncture time was delayed even in the HERS group. Such delays can be attributed to several reasons, including determination of the indications for endovascular treatment by emergency physicians, administration of tPA intravenously before endovascular treatment, and the on-call availability of endovascular physicians at night and on holidays. Second, this was a single-center observational study with a small number of cases and no adjustments for confounding factors. Prospective multicenter studies incorporating a large number of patients will be important to expand the coverage of the HERS and optimize treatment strategies for patients with AIS.

In conclusion, our study demonstrates the potential of the HERS in reducing the treatment time for patients with AIS undergoing endovascular therapy. Although the lack of improvement in neurological outcomes highlights the need for further research and optimization of treatment strategies, the significant reduction in door-to-puncture and door-to-recanalization times suggests that the HERS could be a valuable tool for the management of AIS. As the field of AIS treatment continues to evolve, investigating the effectiveness of the HERS in various patient subgroups and developing comprehensive stroke care protocols that maximize the benefits of this innovative system will become more important.

## Data availability statement

The raw data supporting the conclusions of this article will be made available by the authors, without undue reservation.

## Ethics statement

The studies involving humans were approved by the Institutional Review Board of Jichi Medical University Saitama Medical Center. The studies were conducted in accordance with the local legislation and institutional requirements. The Ethics Committee/Institutional Review Board waived the requirement of written informed consent for participation from the participants or the participants’ legal guardians/next of kin because the requirement for informed consent was waived because of the observational study design, which posed minimal risk to the patients and preserved their anonymity. Patients and their respective families were provided with an opportunity to opt out of the study.

## Author contributions

MK: Conceptualization, Formal analysis, Investigation, Methodology, Project administration, Visualization, Writing – original draft, Writing – review & editing. CN: Data curation, Formal analysis, Supervision, Visualization, Writing – review & editing. YK: Supervision, Writing – review & editing. KT: Writing – review & editing. HT: Supervision, Writing – review & editing. HY: Conceptualization, Supervision, Writing – review & editing. MI: Supervision, Writing – review & editing. KY: Supervision, Writing – review & editing. YY: Supervision, Writing – review & editing. TM: Supervision, Writing – review & editing.
